# Effect of Sivelestat in the Treatment of Acute Lung Injury and Acute Respiratory Distress Syndrome: A Systematic Review and Meta-Analysis

**DOI:** 10.1007/s44231-023-00032-9

**Published:** 2023-06-01

**Authors:** Qiongli Ding, Yi Wang, Chunbo Yang, Dilireba Tuerxun, Xiangyou Yu

**Affiliations:** 1 Critical Medicine Center, the First Afiliated Hospital of Xinjiang Medical University, Urumqi, 830054 Xinjiang Uygur Autonomous Region China; 2 Xinjiang Medical University, Urumqi, 830054 Xinjiang Uygur Autonomous Region China; 3 Xinjiang Uygur Autonomous Region Institute of Critical Medicine, Urumqi, 830054 Xinjiang Uygur Autonomous Region China

**Keywords:** Sivelestat, Acute lung injury, Acute respiratory distress syndrome, Meta-analysis

## Abstract

**Background:**

The efficacy of neutrophil elastase inhibitor sivelestat in the treatment of acute lung injury (ALI) and acute respiratory distress syndrome (ARDS) remains controversial. A systematic review and meta-analysis were performed in accordance with the PRISMA guidelines assess the effect of sivelestat on ALI/ARDS patients, different studies were included.

**Methods:**

Electronic databases, National Knowledge Infrastructure (CNKI), Wan fang data, VIP, PubMed, Embase, Springer, Ovid and the Cochrane Library were searched using the following key words: (“Sivelestat” OR “Elaspol”) AND (“ARDS” OR “adult respiratory distress syndrome” OR “acute lung injury”). All databases published from January 2000 to August 2022. The treatment group was treated with sivelestat and the control group was given normal saline. The outcome measurements include the mortality of 28–30 days, mechanical ventilation time, ventilation free days, intensive care unit (ICU) stays, oxygenation index (PaO_2_/FiO_2_) on day 3, the incidence of adverse events. The literature search was conducted independently by 2 researchers using standardized methods. We used the Cochrane risk-of-bias tool to assess the quality of the included studies. Mean difference (MD), Standardized mean difference (SMD) and relative risk (RR) were calculated using random effects model or fixed effects model. All statistical analyses were performed using RevMan software 5.4.

**Results:**

A total of 2050 patients were enrolled in 15 studies, including 1069 patients in treatment group and 981 patients in the control group. The results of the meta-analysis showed that: compared with the control group, sivelestat can reduce the mortality of 28–30 days (RR = 0.81, 95% CI = 0.66–0.98, *p* = 0.03) and the incidence of adverse events (RR = 0.91, 95% CI = 0.85–0.98, *p* = 0.01), shortened mechanical ventilation time (SMD = − 0.32, 95% CI = − 0.60 to − 0.04, *p* = 0.02) and ICU stays (SMD = − 0.72, 95% CI = − 0.92 to − 0.52, *p* < 0.00001), increased the ventilation free days (MD = 3.57, 95% CI = 3.42–3.73, *p < *0.00001) and improve oxygenation index (PaO_2_/FiO_2_) on day 3 (SMD = 0.88, 95% CI = 0.39–1.36, *p* = 0.0004).

**Conclusions:**

Sivelestat can not only reduce the mortality of ALI/ARDS patients within 28–30 days and the incidence of adverse events, shorten the mechanical ventilation time and ICU stays, increase ventilation free days, but also improve the oxygenation index of patients on days 3, which has a good effect on the treatment of ALI/ARDS. These findings need to be verified in large-scale trials.

## Background

Acute lung injury (ALI) and acute respiratory distress syndrome (ARDS) are clinical syndrome of diffuse inflammatory injury to the lung parenchyma caused by pathological factors such as infection, shock, and trauma. It is one of the main reasons for patients transferred to an intensive care unit (ICU) and its fatality rate is as high as 40% [1]. Decreased lung volume, decreased pulmonary compliance, and severe ventilation/blood flow ratio imbalance are common pathophysiological features of ARDS. Progressive hypoxemia and respiratory distress are the main clinical manifestations of ARDS. There are significant differences in ALI/ARDS caused by different injury factors, and the essence of the currently accepted pathogenesis is the uncontrolled inflammatory response. Neutrophils that are widely activated by the body in an inflammatory state can migrate to lung tissue and release large amounts of proteases such as neutrophil elastase (NE), Cathepsin G (CG), protease 3 (PR3), and neutrophil serine protease 4 (NSP4) [2]. NE is not only an important damage molecule associated with ALI/ARDS, but also can promote the production of neutrophil chemokines and aggravate the inflammatory response. The overexpression of NE causes neutrophils to exude a large number of blood vessels and concentrate on the inflammatory site and release inflammatory factors such as IL-8 and TNF-α, while also degrading the cell base quality, catalyzing caspase3-induced apoptosis, thereby causing damage to the body tissue and matrix. At the same time, it can also enhance the adhesion ability of a variety of viruses and bacteria to human cells, and promote the invasion and metastasis of microorganisms and cancer cells to the human body [3]. Therefore, inhibiting the activity of NE can prevent and mitigate ALI/ARDS.

Sivelestat is a highly specific and systemically active NE inhibitor with a low molecular weight that can reversibly and competitively inhibit neutrophil elastase release, inhibit neutrophil activation and intrapulmonary inflammatory cell infiltration, alleviate the release of inflammatory mediators, thereby improving respiratory function and has a good protective effect on various experimental ALI [4–6]. Early studies in Japan confirmed that sivelestat has the effect of reducing pulmonary vascular permeability [7], inhibiting alveolar epithelial mucus secretion [8], reducing the production of inflammatory cytokines IL-1β, IL-6, and TNF-α [9], protecting lung damage caused by perfusion [10]. Although sivelestat has been approved in Japan for the treatment of ALI with systemic inflammatory response syndrome (SIRS), while the efficacy of sivelestat therapy on outcomes in patients with ALI/ARDS remains controversial. Therefore, a systematic review and meta-analysis of available studies were conducted to evaluate the treatment effect of sivelestat.

## Materials and Methods

### Data Sources, and Search Strategy

This systematic review and meta-analysis were conducted in accordance with the Preferred Reporting Items for Systematic Reviews and Meta-Analyses guidelines. All data were gathered from publicly available sources. In August of 2022, we searched PubMed, Embase, Springer, Ovid, Wan fang data, CNKI and the Cochrane Library for articles reporting the effect of sivelestat on ALI/ARDS patients. Using the following key words: (“sivelestat” OR “elaspol”) AND (“ARDS” OR “adult respiratory distress syndrome” OR “acute respiratory distress syndrome” OR “noncardiogenic pulmonary edema” OR “respiratory insufficiency” OR “systemic inflammatory response syndrome” OR “shock lung” OR “respiratory failure” OR “lung injury” OR “septic shock” OR “sepsis”). Manual searches of the reference lists were also conducted from all relevant original and review articles to identify additional eligible studies. No language restriction was applied. Unpublished trials were excluded. The medical subject heading, methods, patient disease status, study design, intervention, and outcome variables were used to identify relevant studies. The literature search was conducted independently by two researchers using standardized methods. Any inconsistencies are resolved through panel discussions until consensus is reached and included in the meta-analysis. The ethical approval and written consent are not necessary for the meta-analysis, because the data of meta-analysis is collected from published literature.

### Inclusion and Exclusion Criteria

The population, intervention, comparator, outcome and study design (PICOS) approach were used to establish the selection criteria for our systematic review and meta-analysis. We excluded incomplete data or missing data. Studies meeting the following criteria were included: (1) Population: patients with ALI/ARDS. (2) Intervention: sivelestat use. (3) Comparator: The sivelestat group versus the non-sivelestat group. (4) Outcome: acute physiological and chronic health (APACHE II) score, the mortality within 28–30 days, the incidence of adverse events, mechanical ventilation time, ventilation free days, intensive care unit (ICU) stays, and oxygenation index (PaO_2_/FiO_2_) level on day 3 and on day 7. (5) Study Design: we selected randomized controlled trials (RCTs), prospective cohort studies and, due to the scarcity of studies, also included retrospective cohort studies and matched case-control series.

Studies were excluded if they were not written in the English language or if the full text was not available. When the outcomes of studies were reported in multiple publications, the publication with the largest patient cohort with relevant outcome data was included in this review. Results from case reports, reviews, registry data, abstracts, and replies/commentaries were excluded.

### Data Collection and Quality Assessment

The data collected included the first author’s name, publication year, country, sample size, mean age, disease status, intervention, baseline PaO_2_/FiO_2_ ratio, baseline acute physiology and chronic health evaluation (APACHE II) score, reported endpoints, and study design variables. The quality of studies was evaluated in four ways, and the Jadad score ≥3 was included in the meta-analysis according to the Jadad Score Scale.

### Statistical Analysis

All statistical analyses were performed using RevMan software 5.4 (The Cochrane Collaboration, Oxford, UK). For outcome analyses, both random and fixed effect models were used to obtain risk ratios (RRs) with 95% confidence intervals (CIs) for dichotomous outcomes. Forest plots were created using the random-effects model. For continuous outcomes, we extracted the reported means and evaluated the mean difference using both the random and fixed effect models, with the forest plot created with the random-effects model. Heterogeneity was assessed using Cochran’s *Q* test (*p* < 0. 10) and the *I*^2^ statistic. If *I*^2 ^> 50%, heterogeneity was deemed considerable. *p* values < 0.05 were considered statistically significant unless otherwise specified.

## Results

### Study Selection and Study Characteristics

**Fig. 1:**
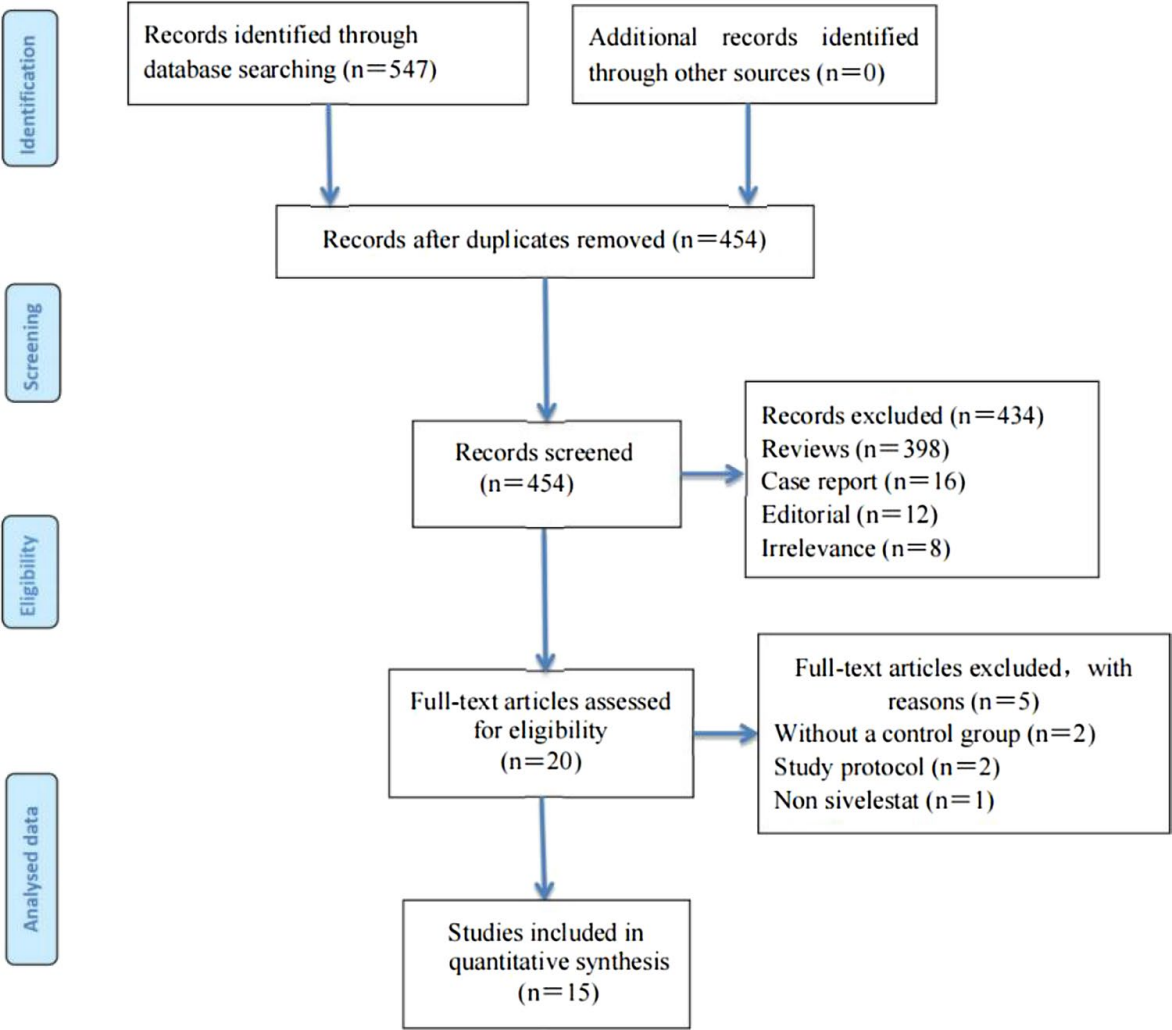
Flow diagram for selection of studies

547 records were identified through a computerized literature search, among which 93 were duplicates and 434 were excluded after an initial review of titles and abstracts. The remaining 20 publications were reviewed in full-text and assessed against inclusion criteria. Finally, 15 studies [11–25] were included in our study. 6 [11–15, 24] studies were classified as RCTs, 8 [16–23, 25] as retrospective cohort studies, and 1[19] as prospective cohort studies. The search and selection process were depicted in a PRISMA flow diagram (Fig. 1). Descriptions of included studies are presented in Table 1. This study included 2050 patients ( 1069 in the sivelestat group and 981 in the control group). Patients in the included studies were routinely actively treated with the primary disease and given symptomatic treatment after diagnosis.

**Table 1:** Baseline characteristic of studies included in the systematic review and meta−analysis

Study	Region	Group	Cases	Years	Disease status	Intervention	APHACHE II score	Outcome indicator
1. Kadio2004 [11]	Japan	S Group C Group	12 12	66 (7) 62 (9)	ARDS	0.20 mg/kg/h	19.9 (3.8) 20.2 (4.0)	①②④⑤
2.Zeiher2004 [12]	Multiple countries	S Group C Group	241 246	56.2 (17.2) 55.8 (17.5)	ALI	0.16 mg/kg/h	21.1 (7.2) 20.5 (6.8)	①②⑤⑥
3.Tamakuma2004 [13]	Japan	S Group C Group	113 108	59.5 (12.9) 56.1 (12.4)	ALI	0.20 mg/kg/h	NR NR	①③⑥
4.Shiral2006 [14]	Japan	S Group C Group	19 16	48.9 (15.4) 46.5 (13.2)	ALI	0.20 mg/kg/h	NR NR	①③④⑥
5.Endo2006 [15]	Japan	S Group C Group	13 13	NR NR	ARDS /ALI	0.20 mg/kg/h	NR NR	①
6.Okayama2006 [16]	Japan	S Group C Group	12 13	73.1 (6.95) 70.4 (7.65)	ARDS	0.20 mg/kg/h	21.9 (3.4) 21.2 (3.2)	①②④
7.Ono2007 [17]	Japan	S Group C Group	7 10	60.7 (11.8) 69.5 (6.7)	ARDS	0.20 mg/kg/h	NR NR	②④
8.Hayakawa2010 [18]	Japan	S Group C Group	34 133	59.4 (20.3) 54.1 (21.2)	ARDS	0.20 mg/kg/h	23.2 (13.4) 20.5 (12.4)	①
9. Hayashida2011 [19]	Japan	S Group C Group	23 21	71.7 (16.8) 63.6 (24.7)	ALI	0.20 mg/kg/h	22.8 (8.5) 23.1 (9.6)	①③④
10.Morimoto2011 [20]	Japan	S Group C Group	10 12	72 (7) 74 (7)	ALI	0.20 mg/kg/h	NR NR	①②④⑤
11.Aikawa2011 [21]	Japan	S Group C Group	374 168	66.1 (14.4) 70.2 (14.4)	ALI	0.20 mg/kg/h	21.3 (7.3) 23.3 (7.9)	③⑥
12.Tsuboko2012 [22]	Japan	S Group C Group	34 15	73(9) 69 (17)	ARDS /ALI	0.20 mg/kg/h	22 (7) 21 (4)	①②④
13.Gao2021 [23]	China	S Group C Group	60 80	56. 2 (15.2) 57.9 (13.0)	ARDS	300 mg/d	9.18 (1.2) 13.1 (1.6)	①②④
14.Zhou2022 [24]	China	S Group C Group	40 40	65.6 (11.2) 65.6 (10.1)	ARDS	0.20 mg/kg/h	NR NR	①②
15.Gu2022 [25]	China	S Group C Group	77 94	51.1 (11.7) 49.2 (10.8)	ALI	0.20 mg/kg/h	19.4 (4.9) 18.4 (4.5)	①③

S group: sivelestat group, C group: control group. NR: not reported. Outcome indicator: ① the mortality of 28-30 days, ②mechanical ventilation time , ③ventilation free days , ④ICU stays, ⑤ oxygenation index (PaO_2_/FiO_2_) on day 3, ⑥the incidence of adverse events

### Meta-analysis Outcomes

#### Publication Bias and Sensitivity Analyses

**Fig. 2:**
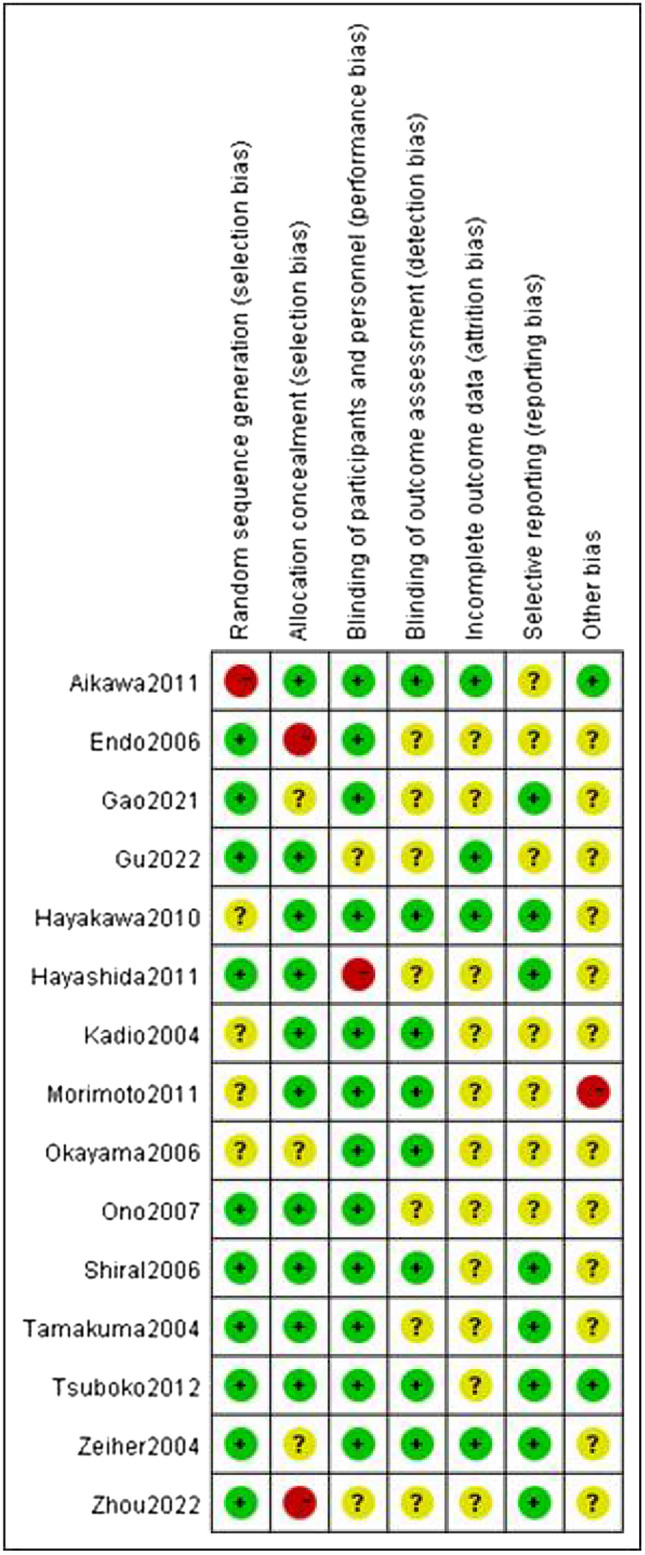
Risk of bias summary

**Fig. 3:**
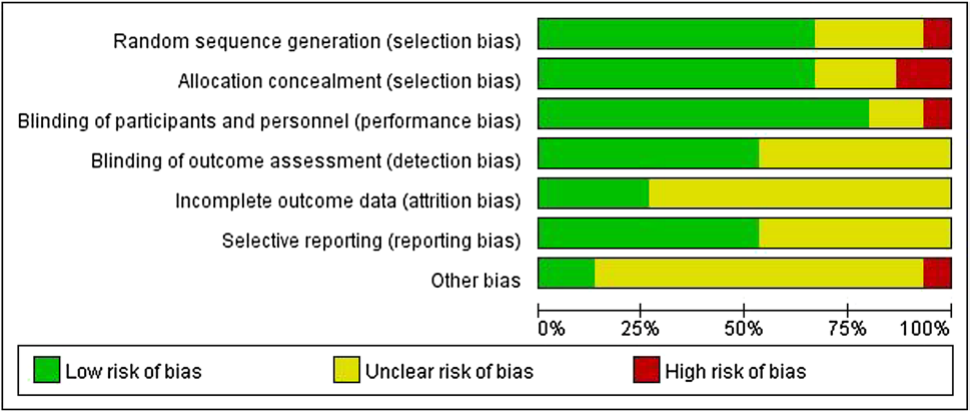
Risk of bias graph

Of the 15 studies [11–25] included, 2 studies [12, 21] were multicenter studies and the remaining 13 [11, 13–20, 22–25] were single-center studies. 4 studies had a high-risk bias factor, because deaths were not reported between the intervention and control groups, loss of follow-up bias was considered a high risk in two studies. All studies were defined as unclear biases because they failed to elaborate in the article. The methodology of 5 studies proposed group-randomization allocation of ALI/ARDS patients included in the study. All studies results data are reported completely, and the quality evaluation of the studies is shown in Figs. 2 and 3.

#### sivelestat Group vs. Control Group

##### The Mortality of 28–30 Days

**Fig. 4:**
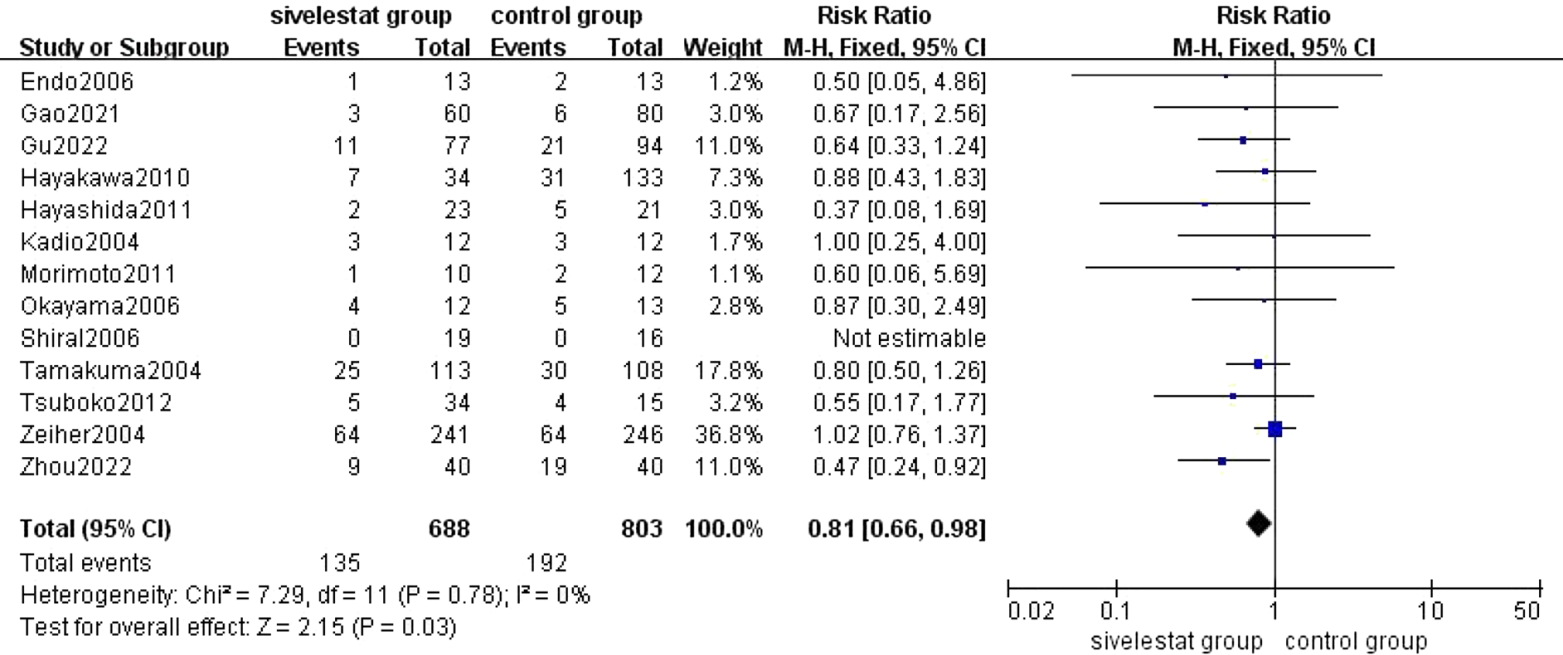
Forest plot for the mortality of 28–30 days

13 [11–16, 18–20, 22–25] studies reported the mortality of 28–30 days in patients with ALI/ARDS in the sivelestat group and the control group. The pooled results from the Fixed effect models for 28–30 days mortality was shown in Fig. 4. A total of 1491 patients were included in the analysis. Of the 327 deaths among 1491 patients with ALI/ARDS, 136 deaths occurred in 688 patients (19.8%) of the sivelestat group, whereas 192 deaths occurred in 803 patients (23.9%) of the control group. Overall analysis of the 13 studies showed that sivelestat significantly reduced 28–30 days mortality in patients with ALI/ARDS compared with the control group (RR=0.81, 95% CI = 0.66–0.98, *p *= 0.03), without substantial heterogeneity (*I*^2 ^= 0%, *p* = 0.78).

##### Mechanical Ventilation Time

**Fig. 5:**
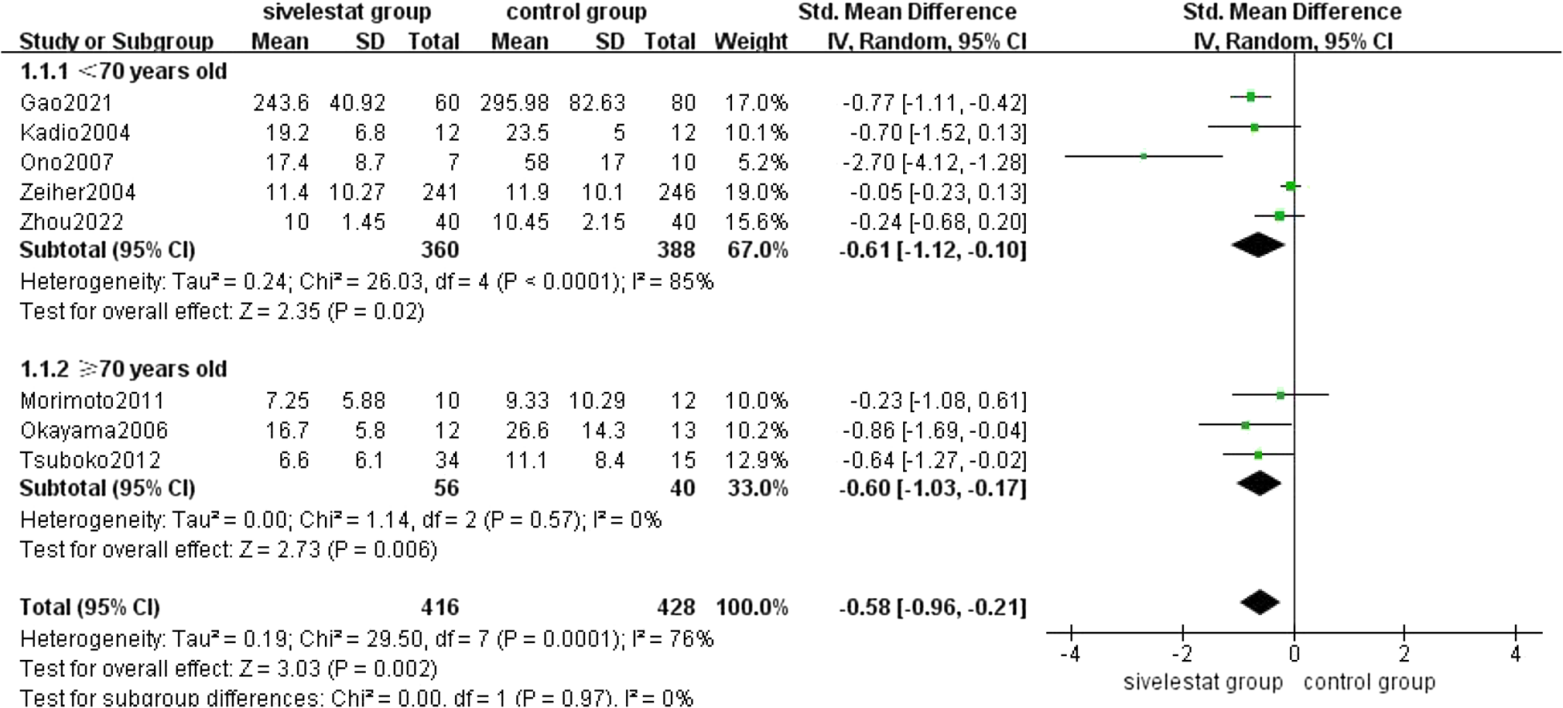
Forest plot for mechanical ventilation time in ALI/ARDS patients of different ages

**Fig. 6:**
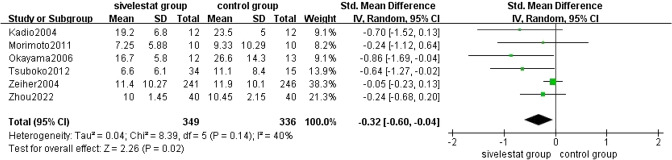
Sensitivity analysis for mechanical ventilation time

8 [11, 12, 16, 17, 20, 22–24] studies provided mechanical ventilation time for patients with ALI/ARDS in the sivelestat treatment and control groups. The pooled results from the Random effect models for mechanical ventilation time was shown in Fig. 5. A total of 844 patients were included in the analysis. Subgroup analyses were performed of patients with ALI/ARDS in the included studies by age greater than 70 years. The results showed that sivelestat significantly reduced mechanical ventilation time in patients <70 years old with ALI/ARDS compared with the control group (SMD = − 0.61, 95% CI = − 1.12 to − 0.1, *p* = 0.02), with substantial heterogeneity (*I*^2 ^= 85%, *p* < 0.0001). And reduced mechanical ventilation time in patients ≥ 70 years old with ALI/ARDS compared with the control group (SMD = − 0.60, 95% CI = − 1.03 to − 0.17, *p* = 0.006), without substantial heterogeneity (*I*^2^ = 0%, *p* = 0.57). The overall heterogeneity of the two different age stages was (*I*^2^ = 76%, *p* = 0.0001). It shows that siveletat can reduce the mechanical ventilation time in patients with ARDS of different ages, but heterogeneity may not be due to age differences. Sensitivity analysis: 8 [11, 12, 16, 17, 20, 22–24] studies involved in evaluating the duration of mechanical ventilation were heterogeneous, and two [17, 21] of them had a greater effect on the pooling effect. After elimination, the heterogeneity decreased significantly (*I*^2^=0.40), and the recalculation effect (SMD = − 0.32, 95% CI = − 0.60 to − 004, *p* = 0.02, Fig. 6). The results did not flip, indicating that the results were stable.

##### Ventilation Free Days

**Fig. 7:**
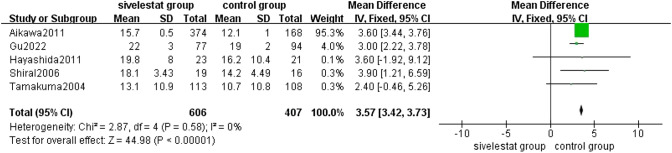
Forest plot for ventilation free days

5 [13, 14, 19, 21, 25] studies provided ventilation free days in patients with ALI/ARDS in the sivelestat group and control groups. The pooled results from the Fixed effect models for ventilation free days was shown in Fig. 7. A total of 1013 patients were included in the analysis. Overall analysis of the five studies showed that sivelestat significantly increased ventilation free days in patients with ALI/ARDS compared with the control group (MD = 3.57, 95%CI=3.42 to 3.73, *p* < 0.00001), without substantial heterogeneity(*I*^2 ^= 0%, *p *= 0.58).

##### ICU Stays

**Fig. 8:**
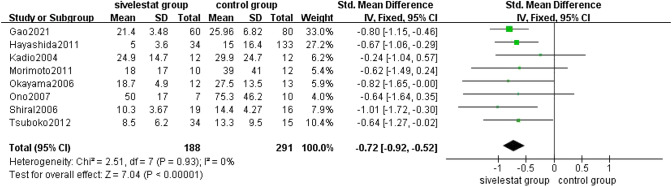
Forest plot for ICU stays

8 studies [11, 14, 16, 17, 19, 20, 22, 23] provided ICU stays in patients with ALI/ARDS in the sivelestat group and control groups. The pooled results from the Fixed effect models for ICU stays was shown in Fig. 8. A total of 479 patients were included in the analysis. Overall analysis of the 8 studies showed that sivelestat significantly shorted ICU stays in patients with ALI/ARDS compared with the control group (SMD = − 0.72, 95% CI = − 0.92 to − 0.52, *p* < 0.00001), without substantial heterogeneity (*I*^2 ^= 0%, *p* = 0.93).

##### PaO_2_/FiO_2_ on Day 3

**Fig. 9:**
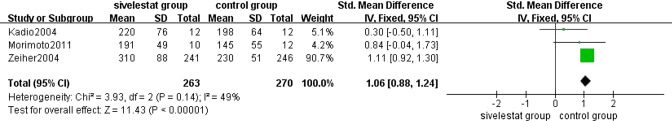
Forest plot for PaO_2_/FiO_2_ on day 3

3 [11, 12, 20] studies reported PaO_2_/FiO_2_ on day 3 in patients with ALI/ARDS in the sivelestat group and the control group. The pooled results from the Fixed effect models for PaO_2_/FiO_2_ on day 3 was shown in Fig. 9. A total of 533 patients were included in the analysis. Overall analysis of the three studies showed that PaO_2_/FiO_2_ on day 3 in the sivelestat group was higher compared with the control group (SMD = 1.06, 95% CI = 0.88 to 1.24, *p* < 0.00001), without substantial heterogeneity (*I*^2 ^= 49%, *p* = 0. 14).

##### The Incidence of Adverse Events

**Fig. 10:**
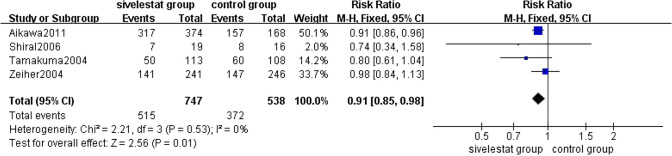
Forest plot for the incidence of adverse events

4 [12–14, 21] studies reported the incidence of adverse events in patients with ALI/ARDS in the sivelestat group and the control group. The pooled results from the Fixed effect models for 28–30 days mortality was shown in Fig. 10. A total of 1285 patients were included in the analysis. Of the 887 adverse events occurred among 1285 patients with ALI/ARDS, 515 adverse events occurred in 747 patients (68.9%) of the sivelestat group, whereas 372 adverse events occurred in 538 patients (69. 1%) of the control group. Overall analysis of the three studies showed that sivelestat significantly reduced the incidence of adverse events in patients with ALI/ARDS compared with the control group (RR = 0.91, 95% CI = 0.85–0.98, *p *= 0.01), without substantial heterogeneity (*I*^2 ^= 0%, *p* = 0.53).

## Dicussion

ARDS is a serious lung disease that can cause severe damage to the lungs in patients with various injuries, including pneumonia, sepsis, trauma, burns, or acute pancreatitis. According to statistics, from SARS in 2003, to MERS in 2012, and then to the outbreak of novel coronavirus pneumonia (COVID-19) in the winter of 2019, the incidence of ARDS in these three outbreaks was 20%, 20–30%, and 18–30% [26]. Studies have shown that the pathogenesis of ALI/ARDS is mainly manifested by the aggregation and activation of inflammatory cells, the synthesis and release of inflammatory mediators, the destruction of alveolar surfactants, and neurological factors [27]. In the pathological state, neutrophil activation and endothelial cell adhesion promote neutrophils to secrete elastase and reactive oxygen species (ROS), which will lead to damage to the extracellular matrix and basement membrane of pulmonary vessels, causing pulmonary capillary leakage, pulmonary edema, and eventually affecting lung gas exchange, and eventually dyspnea and hypoxemia [28]. Inhibiting the activity of NE can block alveolar collapse and pulmonary capillary leakage, thereby reducing pulmonary edema and ventilation disorders [29].

In the face of the high incidence of ARDS in the three outbreaks, several ventilation interventions such as tidal volume reduction [30], positive end-expiratory pressure (PEEP) [31], and adjunctive measures such as prone position, neuromuscular block [32], and extracorporeal membrane oxygenation (ECMO) have been proposed [33]. Although treatment strategies such as protective mechanical ventilation techniques have reduced the risk of death in patients with ARDS, current pharmacotherapy for ADRS has been poorly treated for a long time. Sivelestat is a neutrophil elastase inhibitor, which induces competitive inhibition of neutrophils, inhibition of neutrophil activation, and reduction of inflammation in the lungs. This study conducted a systematic review of sivelestat in the treatment of ALI/ARDS, which provided a reliable basis for clinical application.

The results showed that sivelestat therapy might play an important role on the PaO_2_/FiO_2_ level, while it had no significant effect on 28–30 days mortality, ventilation days, and ICU stays [34]. The limitation of these studies are as follows: the number of included studies was smaller than expected. Moreover, the estimation description of the sample size is not detailed, which may lead to insufficient test performance and affect the results. At the same time, the observation indicators of each study varied, and some indicators were only reported in individual studies. Therefore, more studies were included in this study to more fully evaluate the effects of sivelestat in patients with ALI/ARDS. This meta-analysis added ventilation free days, the incidence of adverse events and other outcome indicators, and the conclusions were more comprehensive. The results showed that sivelestat could not only reduce the 28–30 days mortality of ALI/ARDS patients, but also reduce the incidence of adverse events. It can also shorten mechanical ventilation time and ICU stays, increase ventilation free days, and improve PaO_2_/FiO_2_ on days 3, thereby improving the prognosis of patients with ALI/ARDS.

While the prospect of sivelestat in the treatment of ARDS is exciting, many problems remain. The timing of the intervention and the duration of the medication may be key to its ultimate success. Based on previous treatment experience, early intervention appears to increase the chances of success. However, in a 2004 study by Zeiher [12], a number of adverse events occurred, such as allergic reactions, hepatobiliary disease, blood and lymphatic system diseases, and kidney and urinary system diseases. Although the prescribed indications for discontinuation were not met, the increase in deaths in the treatment group over a long period of time forced the DSMB facility to terminate the study. However, the occurrence of adverse events must be confirmed in larger prospective trails and needs to be evaluated over a long follow-up period. Thus, the Kido study extended follow-up in patients with ALI/ARDS, confirmed that patients in the sivelestat treatment group had a lower actual mortality rate at 30, 60, and 90 days than in the group not receiving sivelestat, respectively, by 7.0%, 9. 1%, and 8.9%, and found a higher success rate in young, non-cancerous, non-hemodialysis, non-use of high-dose hormones, and was first proposed in patients receiving sivelestat [35]. The Asian population may have racial precedence, suggesting that sivelestat can be used as a potential therapeutic agent for ALI/ARDS. In 2021, Gao [23] confirmed the efficacy of sivelestat on sepsis-associated ARDS in a study conducted in China and pharmacoeconomically demonstrated that it significantly reduces medical costs for patients. With the application of drugs such as sivelestat, the treatment of ARDS to curb inflammatory overresponse is moving from early non-specific inflammation inhibition to a new era of targeted inhibition.

There are some limitation in this study: (1) only the Chinese and English databases are searched, and only the Chinese and English literature is included, which may lead to language bias; (2) most of the included trials were conducted in Japan, which may have led to racial bias; (3) the lack of uniform ARDS diagnostic criteria, differences in diagnosis in all patients included in the study may lead to different outcomes; (4) the overall quality of the trails included in the analysis was not high, and most of the studies did not describe the randomized method and whether to use double-blindness, and there may be selective bias; (5) although there is no statistical heterogeneity in the study of mortality outcomes and there is no significant difference in relative risk, there may be differences between studies in terms of patient populations. Other treatments, such as ventilation, use of other drugs, or infusions, may alter the outcome, and differences in the etiology of ALI/ARDS may also affect the efficacy of sivelestat.

## Conclusion

In summary, this study shows that sivelestat has a certain efficacy in the treatment of ALI/ARDS, which can not only reduce the 28–30 days mortality of ALI/ARDS patients, but also reduce the incidence of adverse events. It can short mechanical ventilation time and ICU stays, increase ventilation free days, and improve PaO_2_/FiO_2_ on days 3. It is hoped that some high-quality trails will be further validated based on the shortcomings of existing studies, and future large-scale trials should focus on different disease states, patient characteristics, and trials from different countries to explore the safety and efficacy of sivelestat in ALI/ARDS applications.

## Data Availability

The data that support the findings of this study are openly available in National Knowledge Infrastructure (CNKI), Wan fang data, VIP, PubMed, Embase, Springer, Ovid and the Cochrane Library.
